# Scientific Practice

**Published:** 1882-02

**Authors:** 


### SCIENTIFIC PRACTICE.

A noted allopathist has said: "Homœopathy has no success in comparison with the *cautious, scientific practice of to-day!*" We quote a sample of this cautious, scientific practice, and italicize some of its especially *scientific* features. One can scarcely believe that the author was not joking, or insane. The disease was "Trichinosis":

"The treatment I adopted was to meet symptoms, for unfortunately this is one of the diseases whose cause we know, but as yet cannot influence without placing the life of the patient in jeopardy.

"Niemeyer says that benzine has not been given a fair trial, and believes it is efficient, but I could not get any here to try. First of all, I gave calomel to expel what parasites might have remained in the intestinal canal. I administered carbolic acid freely, simply for its antiseptic properties, or, *it may be, because I had a large quantity of it.* On the *same principle* I used salicylic acid. Quinine was given as an antipyretic and antiperiodic (the locality being malarious), and preparations of iron for those who were convalescing. I also gave iodide of potassium, *out of mere curiosity*, and used stimulants but very little, only when indicated by feebleness of the first sound of the heart."

